# Capturing the Molecular and Biological Diversity of High-Grade Astrocytoma in Genetically Engineered Mouse Models

**DOI:** 10.18632/oncotarget.425

**Published:** 2012-01-25

**Authors:** Lionel M.L. Chow, Suzanne J. Baker

**Affiliations:** ^1^ Division of Oncology, Cancer and Blood Diseases Institute, Cincinnati Children's Hospital Medical Center; ^2^ Department of Developmental Neurobiology, St. Jude Children's Research Hospital

**Keywords:** glioblastoma, astrocytoma, mouse model, PI3K pathway

## Abstract

High-grade astrocytoma remains a significant challenge to the clinician and researcher alike. Intense study of the molecular pathogenesis of these tumors has allowed identification of frequent genetic alterations and critical core pathways in this disease. The use of novel mouse genetic tools to study the consequence of specific mutations in brain has led to the development of multiple representative genetically engineered mouse models that provided novel insights into gliomagenesis. As we learn more about the biology of high-grade astrocytoma from the study of these models, we anticipate that our improved understanding will eventually lead to greater success in clinical trials and improved outcome for patients.

## INTRODUCTION

High-Grade Astrocytomas (HGAs) are among the most aggressive and deadly malignancies in man. Unlike most cancers, these primary central nervous system (CNS) tumors do not achieve their lethality by metastatic spread to secondary organs, leading to overwhelming tumor burden and multiorgan failure in patients. The devastating effects of HGAs can be traced to multiple contributing factors. 1) The brain is encased within a solid cranial vault such that progressive tumor expansion leads not only to specific neurological signs and symptoms but also to generalized CNS depression and dysfunction. 2) Tumors are often localized within or adjacent to critical brain structures making complete surgical resection neurologically devastating and therefore impossible. 3) HGAs are highly invasive such that even in cases where complete radiographic resection is achieved local and/or distant microscopic spread of tumor cells at the time of diagnosis usually leads to rapid tumor regrowth. 4) Tumor cells and in particular glioma stem-like cells exhibit a high degree of radio and chemoresistance due to both intrinsic (eg. tumor heterogeneity, rapid mutational adaptation) and extrinsic (eg. blood-brain barrier) mechanisms. As a consequence, despite contemporary multimodal therapy that includes aggressive surgery, maximally delivered radiotherapy and temozolomide, median overall survival for glioblastoma, the most advanced form of HGA, is 15-18 months, a dismal prognosis which has not significantly improved in several decades [1, 2]. It would appear that further incremental improvements in neurosurgery, radiation therapy and conventional chemotherapy will have minimal impact on this disease. Therefore novel therapeutic modalities and agents are desperately required to improve patient outcome.

This persistently bleak clinical outlook for HGA has led to the viewpoint that the traditional laboratory models used to study disease biology and to conduct pre-clinical trials are not adequately representative of the human condition. Concurrently, technical advancements in the manipulation of mouse genetics have resulted in the recent development of several novel genetically engineered mouse models (GEMMs) for HGA. Accumulating evidence suggests that these models faithfully recapitulate the histology and molecular biology of HGA and therefore, they may provide new insights towards understanding the aggressive nature of this malignancy and lead to the development and testing of novel therapeutic strategies. Herein, we review the main concepts that define HGA molecular pathology, present a selection of GEMMs that echo these concepts and outline directions in which translational applications are being applied.

## CORE PATHWAY MUTATIONS IN HIGH-GRADE ASTROCYTOMAS

Gliomas are diffusely infiltrating tumors of the CNS that encompass a spectrum of histologically distinct but overlapping neoplasms. The current World Health Organization (WHO) classification scheme reflects this heterogeneity and segregates tumors according to their grade and predominant histological features [3]. Astrocytic tumors are the largest histological group and can occur at all grade levels from 1 to 4. Grade 1 astrocytoma, also referred to as pilocytic astrocytoma appears to have a molecular etiology unrelated to other astrocytic tumors and behaves clinically as a distinct entity [4]. The remaining grades of astrocytomas lie along a spectrum of histopathology demonstrating progressively more aggressive features that culminate in grade 4 tumors known as glioblastoma [3]. Low grade or diffuse astrocytomas are grade 2 tumors that frequently undergo malignant transformation leading to disease recurrence in patients as HGAs (grade 3 or 4). Less than 10% of glioblastomas arise in this manner, and are termed secondary glioblastomas. Primary glioblastomas comprise the vast majority of glioblastomas, and occur in the absence of an antecedent low grade lesion. Genome-wide and integrative genomic analyses have resulted in the identification of molecularly defined and biologically distinct tumor subgroups.

**Figure 1:**
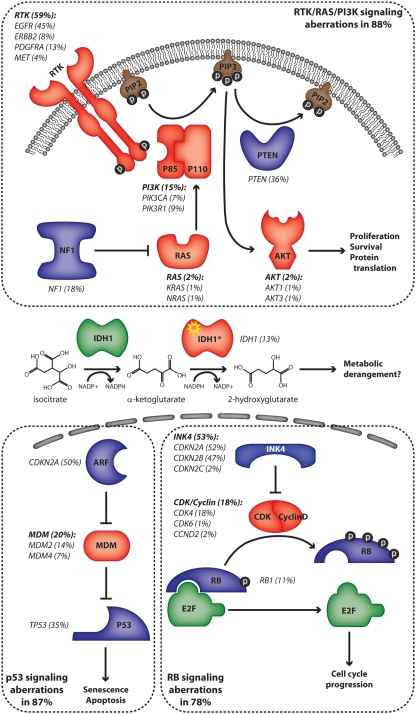
Frequency of pathway mutations in high-grade astrocytoma The frequency of mutations affecting intermediates of several pathways is indicated in parentheses. Proteins whose genes are amplified or have an activating mutation are represented in red while those whose genes are deleted or have an inactivating mutation are in blue. The proteins are grouped according to the core pathways in which they function (dotted lines). The IDH1 mutant proteins (IDH1*) possess a neomorphic catalytic activity which is depicted. The frequencies of mutations are derived from references 14 and 22.

Many frequently mutated tumor suppressor genes and oncogenes in HGA are associated with recurrent chromosomal aberrations. Notable examples include loss of heterozygosity (LOH) and inactivating point mutations of the tumor suppressor genes *TP53* [5, 6] and *PTEN* [7, 8], homozygous deletions of *CDKN2A* [9-11], and amplification with or without activating mutations of the oncogene *EGFR* [12, 13]. More recently, investigators used genome-wide approaches to catalog the genetic changes in HGA [14, 15]. These groundbreaking studies have led to the current concept that most if not all HGAs are characterized by dysregulation of three core pathways: the receptor tyrosine kinase (RTK)/phosphatidylinositol 3'-kinase (PI3K)/AKT axis, p53 signaling and RB-mediated control of cell cycle progression (Figure 1). These studies also validated the importance of *NF1* inactivation in non-syndromic HGA and identified novel players in gliomagenesis, such as *PIK3R1* and *IDH1* whose mechanism of action is the subject of intense investigation [16].

The RTK/PI3K/AKT pathway regulates many aspects of cellular physiology including proliferation, protein translation, cell size, cell survival, cell migration and motility, all of which have been implicated in the malignant phenotype (Figure 1) [17]. The precise outcome of pathway activation is strictly context dependent and it is not entirely clear which of these effects are most important during gliomagenesis. This pathway is targeted for mutational activation at multiple nodes [14, 15]. Amplifications and activating mutations of the RTK genes *EGFR*, *PDGFRA*, *ERBB2* and *MET* are seen in 59% of HGAs. The *EGFR* gene is by far the most commonly activated oncogene in HGA. Another commonly targeted node in this pathway is the production of the secondary messenger molecule phosphatidylinositol (3,4,5)-trisphosphate (PIP3). Three critical genes regulating this process are targeted for mutation: *PIK3CA* and *PIK3R1*, which encode the catalytic and regulatory subunits of Class 1A PI3K respectively, and *PTEN*, the gene encoding the lipid phosphatase that opposes PI3K activity. Together, these genes are mutated in 49% of HGAs. Mutations and homozygous deletions of the *NF1* gene are present in 18% of tumors [14, 15]. NF1 contains a GTP-ase activating protein (GAP) domain and is thought to exert tumor suppressive activity primarily by inactivation of RAS proteins. Interestingly, neither study found significant activating mutations of RAS or RAF family members, which are commonly targeted oncogenes in tumors driven by RAS/MAPK signaling such as pilocytic astrocytoma, lung and colon carcinomas and melanoma [4, 18-20]. This suggests that the primary outcome of *NF1* inactivation may be to boost PI3K/AKT signaling via crosstalk between pathways, which has been demonstrated in glioma GEMMs (see below). Finally, members of the AKT family are infrequently targeted for gene amplifications. Notably, there is an absence of mutations in downstream effectors of AKT suggesting that multiple effectors of the pathway may play critical roles in gliomagenesis. Alternatively, such mutations may have a net deleterious effect on tumor growth due to the existence of feedback inhibition circuits. These are all potentially critical considerations when selecting appropriate inhibitors of the RTK/PI3K/AKT pathway for HGA therapy.

*TP53* is the most commonly mutated tumor suppressor gene in human cancer. The P53 pathway regulates the critical checkpoint that detects oncogenic stress and DNA damage, which if unresolved leads to cellular senescence or apoptosis (Figure 1). In HGA, the *TP53* gene along with genes encoding regulators of p53 stability (*MDM2*, *MDM4* and *CDKN2A* which encodes p19^ARF^) are targeted in at least 87% of cases [14]. Therapeutic strategies aimed at stabilizing p53 via inhibition of MDM2 and/or MDM4 are predicted to be effective in tumors with intact p53 function, therefore characterization of specific pathway mutations is potentially important.

The RB family, and in particular phosphorylation of its primary member RB1, regulates the cell cycle checkpoint at the G_1_/S boundary (Figure 1). The INK4 family of proteins negatively regulates the phosphorylation of RB by suppressing a complex containing D-type Cyclins and the Cyclin-dependent kinases CDK4 or CDK6. While *RB1* itself is deleted or mutated in 11% of HGAs, its upstream regulation is more frequently targeted with *CDKN2A* and *CDKN2B*, which encode INK4A and INK4B respectively, being homozygously deleted in 47% of tumors [14]. CDK inhibitors under development would not be expected to have an effect on tumors in which *RB1* or downstream effectors are mutated.

Using an unbiased approach to sequence nearly all of the protein-coding exons in the genome, Parsons *et al*. identified novel mutations in the *IDH1* gene in 11% of HGAs (Figure 1) [15]. Strikingly, these tumors were all secondary glioblastomas and the presence of mutations was associated with a better overall outcome. Subsequent characterizations of much larger cohorts of patients have confirmed these associations and clearly implicate *IDH1* mutation and to a lesser extent mutations in the mitochondrial isoform, *IDH2*, as an early event in gliomagenesis [21, 22]. Interestingly, the overwhelming majority of mutations occur at a single amino acid residue (R132 in IDH1 and R172 in IDH2) which appears to confer a neo-catalytic activity to these proteins. While the native enzyme catalyzes the oxidative decarboxylation of isocitrate to α-ketoglutarate, the mutant form is able to catalyze the reduction of α-ketoglutarate to 2-hydroxyglutarate in an NADPH-dependent process [23, 24]. At present, the mechanism of mutant IDH1-initiated gliomagenesis is not known, however the consequences of its neo-catalytic activity to cellular metabolic pathways that may promote oncogenesis have been discussed [25, 26].

## GENE EXPRESSION SIGNATURES IN HIGH-GRADE ASTROCYTOMAS

Whole transcriptome analysis has emerged as an important method to compare tumor samples and may lead to improved patient stratification and determination of prognosis. This method was used to define expression signatures that distinguish HGA patients according to differential survival outcomes in two independent studies [27, 28]. In both cases, a subgroup of tumors, termed Proneural, expresses genes associated with neurogenesis and was found to have a significantly better survival compared to the remaining tumors. Poor outcome tumors can be subdivided into at least two groups: one expressing markers of angiogenesis and mesenchymal cells (Mesenchymal subgroup) while another expresses an abundance of genes associated with mitosis and proliferation (Proliferative subgroup). Building on these results, Verhaak *et al*. analyzed the multiplatform glioblastoma data from the Cancer Genome Atlas Research Network [29]. Unsupervised gene expression analysis identified similar Proneural and Mesenchymal HGA subgroups along with two other subgroups which they termed Neural and Classical. A recent comparison of subgroups defined by these two approaches demonstrated that the subgroups with the strongest identity were the Proneural and Mesenchymal signatures [30]. Integrated analysis of gene expression signatures and genomic mutation data [14] showed that the majority of tumors with *IDH1* mutations as well as *PDGFRA* amplifications and activating mutations clustered with the Proneural subgroup [29]. In contrast, *NF1* mutations, LOH and loss of expression were more highly associated with Mesenchymal tumors. Finally, the Classical subgroup was characterized by frequent amplifications and mutations of the *EGFR* gene (including the *EGFRvIII* variant) and strikingly, by the absence of *TP53* mutations. The mutual exclusivity of HGAs with PDGFR-α expression, EGFR expression and loss of NF1 was also seen in a targeted proteomic study [31].

Ultimately, the goals of genome-wide and integrative genomic analyses are to provide a template for accurate molecular classification of HGA that will be highly predictive for prognosis and can be used to stratify patients for optimal therapy.

## PEDIATRIC HIGH-GRADE GLIOMAS ARE RELATED BUT MOLECULARLY DISTINCT FROM ADULT HGA

While the same WHO criteria apply to the classification of pediatric high-grade gliomas (HGGs), several differences in the clinical behavior of patients hint at fundamental differences that may exist in tumor biology. 1) Malignant transformation of low-grade astrocytomas in children has been described but appears to occur at a much lower frequency than observed in adult patients [32]. 2) A significant proportion of pediatric HGGs occur in the brainstem (diffuse intrinsic pontine glioma) or in the thalamus (bithalamic glioma) [4], locations rarely encountered in adults. 3) The addition of temozolomide to standard irradiation confers a survival advantage albeit modest to adults with HGA while this does not appear to be the case in children [33]. Directed analyses of genes commonly mutated in adult HGA revealed that mutations and losses of *TP53* [34] and *CDKN2A* [35] occur with relative frequency while mutations and losses of *PTEN* and in particular amplifications of the *EGFR* gene are rare in pediatric HGG [36]. These observations were largely corroborated in large genome-wide analyses of chromosomal aberrations [37-40]. The most common focal amplification and deletion respectively were high-level amplification of *PDGFRA* (14%) and homozygous deletion of *CDKN2A* (16%). *IDH1* hotspot mutations are not common in pediatric HGG [39, 41]. Overall, unlike adult tumors there is a lack of high frequency genetic targets in pediatric HGG.

Unsupervised gene expression analysis identified three subgroups corresponding to the Proneural, Proliferative and Mesenchymal subclasses seen in adult HGA [39]. However, with the exception of *PDGFRA* amplifications which clustered in the Proliferative subgroup (and not in the Proneural group as in adults) there was little correlation between focal genetic aberrations and specific subgroups. Furthermore, copy number alterations targeting genes within core pathways were clearly present in pediatric HGG at lower frequency than in adult tumors. This suggests that these tumors utilize alternative mechanisms to dysregulate the pathways or that core pathways play a diminished role in pediatric HGG. Collectively, these studies show similar gene expression-defined subgroups in pediatric and adult HGGs that are driven by a different set of low-frequency genetic events in children.

## MODELING CORE PATHWAY MUTATIONS IN HGA

As our understanding of genetic abnormalities underlying HGAs becomes more comprehensive, increasingly complex GEMMs have been developed to investigate the contribution and interplay between driver mutations (Table 1). Several models have addressed the relative contribution of core pathways in gliomagenesis. By using an inducible *GFAP-CreER* conditional mouse line that drives *loxP*-dependent recombination in astrocytes and adult neural precursors, cooperativity between the three tumor suppressor genes, *Trp53*, *Pten* and *Rb1*, for gliomagenesis was explored in the context of the adult brain [42]. This study demonstrated that deletion of *Pten* alone or in combination with *Rb1* was unable to initiate glioma formation. In contrast, *Trp53* deletion in conjunction with loss of the *Pten* or *Rb1* tumor suppressor gene resulted in high frequency HGA, while deletion of *Trp53* alone resulted in HGA with low frequency and with prolonged latency. The importance of concurrent dysregulation of the three core pathways was confirmed as HGAs resulting from targeted deletion of *Trp53* and *Pten* acquired secondary mutations in Rb pathway genes while Pi3k/Akt pathway activation was seen in tumors arising from *Rb1* and *Trp53* conditional ablation. Accordingly, mice engineered with targeted deletion of all three tumor suppressor genes developed HGA with a significantly reduced latency.

**Table 1:** Comparison of genetically engineered mouse models

Cre Driver Line/Viral Driver Construct	Targeted Genes	Tumor Histology	Core Pathway Mutation/Dysregulation	Tumor Initiating Cell	Tumor Location	Expression Subgroups	Reference
*GFAP-CreER* (induction at 4wk)	*Pten, Trp53*	HGA	mutation	adult NPC or astrocyte	proliferative and non-proliferative zones	PN, Prolif, Mes	42
	*Pten, Trp53, Rb1*	HGA	mutation				
	*Rb1, Trp53*	HGA, PNET, ONB	dysregulation			NA	
Adeno-*Cre* (injected into lateral ventricle)	*Pten, Trp53*	HGA	dysregulation	postnatal SVZ cells	SVZ	NA	43
	*Pten, Trp53, Rb1*	PNET	NA				
	*Rb1, Trp53*	PNET	NA				
*GFAP-Cre*	*Trp53*	HGA, MB	dysregulation	early NPC or OPC	proliferative zones	NA	46
Adeno-*Cre* (injected into striatum at 3mo)	*EGFR VIII, Cdkn2a, Pten*	HGA	NA	glial cells	striatum	NA	47
RCAS-*PDGFB* +/− RCAS-*Cre* (injected into SVZ, cortex or cerebellum of *Nestin-tv*-a mice at 4wk)	*PDGFB, Cdkn2a, Pten*	HGA	NA	nestin positive cells	proliferative and non-proliferative zones	NA	48
*GFAP-Cre*	*Nf1, Trp53*	HGA	dysregulation	early SVZ cells	SVZ	NA	52
*GFAP-Cre*	*Nf1, Trp53, Pten*	HGA	dysregulation	early SVZ cells	SVZ	NA	51
*Nestin-CreER* (induction at 4wk)	*Nf1, Trp53*	HGA	NA	adult NPC	proliferative zones	NA	53
	*Nf1, Trp53, Pten*	HGA	NA				
Adeno-*Cre* (injected into SVZ at 4wk)	*Nf1, Trp53*	HGA	NA	adult SVZ cells	SVZ	NA	53
	*Nf1, Trp53, Pten*	HGA	NA				
Retroviral *PDGFB/Cre* (injected into subcortical white matter at 6wk)	*PDGFB, Pten*	HGA	NA	adult OPC	white matter	PN	61
	*PDGFB, Pten, Trp53*	HGA	NA				
*GFAP-Cre*	*Nf1, Trp53*	HGA	NA	early OPC	proliferative zones	PN	63

Abbreviations: SVZ – subventricular zone; HGA – high-grade glioma; PNET – primitive neuroectodermal tumor; ONB – olfactory neuroblastoma; MB – medulloblastoma; NA – not assessed; NPC – neural progenitor cell; OPC – oligodendrocyte progenitor cell; PN – Proneural; Prolif – Proliferative; Mes – Mesenchymal

Using a different approach to target tumor suppressor genes for deletion in post-natal neural progenitor cells resulted in divergent results. Cre-expressing adenovirus was directly injected into the lateral ventricle of mice carrying various combinations of floxed tumor suppressor gene alleles [43]. Only the combination of *Trp53* and *Pten* co-deletion resulted in the development of HGA in mouse brains whereas combined deletion of *Trp53* and *Rb1* or all three together generated primitive neuro-ectodermal tumors (PNETs). Interestingly, overexpression of *Cdk4* was present in the *Trp53*; *Pten* deleted HGAs demonstrating that Rb-mediated regulation of cell-cycle progression is a core pathway in these tumors. The selective advantage for the development of PNET in this setting likely reflects a less specific manner in which cells are targeted for Cre-mediated recombination. Indeed, accumulating experimental evidence indicates that different types of brain tumors, and even different subgroups within a histological diagnosis, arise as a consequence of specific driver mutations occurring in specific susceptible cell populations [44, 45]. It follows that for relatively promiscuous driver mutations such as loss of *Trp53*, *Rb1* or *Pten*, the cell being targeted as well as the developmental context in which mutations occur, are likely to be critical determinants of the resulting tumor type.

The importance of developmental context is apparent in a study in which an intragenic deletion of *Trp53* in embryonic neural stem cells was mediated by a *GFAP-Cre* transgene [46]. HGAs were primarily observed however, a discernible fraction of tumors that developed were medulloblastomas, likely arising from progenitor cells targeted in the developing mouse brain. In agreement with the two previously mentioned studies the HGAs described in these mice also demonstrated dysregulation of the Rb and RTK/Pi3k/Akt pathways.

The contributions of various intermediates of the RTK/Pi3k/Akt pathway have been explored in GEMMs. Consistent with the finding that *Pten* deletion alone does not lead to gliomagenesis, activation of other pathway intermediates on their own is similarly inefficient at initiating HGAs. Neither virally-induced expression of the activated EGFR variant, EGFRvIII [47], nor PDGF-B in adult neural progenitor cells [48] was sufficient on their own to initiate HGA. However, when coupled with germ-line deletion of *Cdkn2a* and deletion of *Pten* in the striatum, EGFRvIII expression resulted in robust tumor formation [47]. Likewise, germ-line deletion of *Cdkn2a* or *Trp53* coupled with expression of PDGF-B efficiently initiated HGAs in adult mice [48]. Underscoring the differences between adult and pediatric tumor biology, when PDGF-B was expressed by targeted viral transduction into neonatal neural progenitors, many mice developed low grade gliomas while a small number harbored HGGs [49]. The proportion and aggressiveness of HGGs were greatly enhanced by germ-line loss of the *Cdkn2a* gene but not by *Trp53* deletion.

The role of the *Nf1* tumor suppressor gene in gliomagenesis has also been investigated using GEMMs. Conditional ablation of the gene in the developing brain using a *GFAP-Cre* driver mouse line was insufficient to initiate gliomagenesis [50] however, when paired with germ-line or conditional deletion of *Trp53*, malignant gliomas were efficiently produced [51, 52]. As in the other models discussed, tumors in this context demonstrated Rb pathway dysregulation as demonstrated by robust expression of Cdk4 and Cyclin D1 [52]. Interestingly, the Pi3k/Akt pathway was significantly activated in Nf1-null HGAs implying crosstalk between these downstream RTK effectors. Furthermore, loss of the Pten tumor suppressor enhanced gliomagenesis in this model [51]. These investigators subsequently showed that deletion of *Nf1* and *Trp53* with or without Pten loss in adult neural progenitors gave rise to HGAs [53].

Collectively, these results support the notion that three core pathways play critical roles in human and mouse HGA. The experimental evidence that loss of p53 function, but not Pten or Rb, contributes to early stages of gliomagensis in mice is consistent with the elevated incidence of astrocytomas in individuals with Li-Fraumeni syndrome but neither in Pten Hamartoma Tumor Syndrome (PHTS) nor in patients with germline *RB1* mutations [54-56]. Furthermore, mutations in *TP53*, but not *PTEN* or *RB1*, are found in low-grade astrocytomas prior to malignant transformation [57]. Therefore, loss of *TP53* would appear to be an early mutation in both primary and secondary glioblastoma.

## GEMMS RECAPITULATE MOLECULAR PATHOLOGY OF HGAS

All of the GEMMs described reproduce human histological features characteristic of aggressive HGAs. However, molecularly targeted therapies are now the focus of many clinical HGA trials with the expectation that they will eventually augment or replace our current treatment regimens. It is therefore critically important for preclinical models of the disease to mirror not only the histology of human astrocytomas but also their molecular pathology. To address this, tumors arising from adult mouse brains with targeted deletions of *Trp53* and *Pten* or *Trp53*, *Pten* and *Rb1* were subjected to genome-wide molecular profiling [42]. These analyses revealed striking similarities to human HGAs at both the genomic and transcriptomic levels. Array comparative genomic hybridization (aCGH) identified focal as well as large-scale chromosomal amplifications and losses characteristic of human HGAs. Focal amplifications included those targeting the RTK genes *Met*, *Pdgfra* and *Egfr* and genes encoding the upstream Rb regulators *Cdk4*, *Cdk6* and *Ccnd1*. Meanwhile, gene expression analyses segregated tumors into three subgroups with significant identity to the Proneural, Proliferative and Mesenchymal subgroups defined by human glioblastoma gene expression studies [28, 29].

It is particularly interesting to note that in these murine astrocytomas, multiple mutations within core pathways were frequently observed [42]. RTK gene amplifications were noted in both *Trp53* and *Pten* as well as in *Trp53*, *Pten* and *Rb1* mutated tumors. Importantly, these amplifications appeared to drive maximal signaling through the Pi3k pathway suggesting that ablation of Pten function, the principal negative regulator of Pi3k signaling, is not sufficient to activate the pathway. HGAs with concurrent mutations of *PTEN* and RTK genes have been noted in several studies [14, 58]. Furthermore, several murine astrocytomas were characterized by co-amplifications of more than one RTK gene [42], a phenomenon that has also been appreciated in human tumors [14, 59, 60]. In similar observations, multiple mutations affecting Rb pathway signaling were observed in both mouse [42] and human HGAs [14, 59]. These observations have important implications for the selection of targeted agents and suggest that pathway inhibition at multiple nodes may be required to achieve maximal therapeutic effect.

A recent study employed a retrovirus encoding PDGF-B and the Cre recombinase which was directly injected into the subcortical white matter of adult mice [61]. HGAs arose efficiently in mice in which *Pten* or the combination of *Pten* and *Trp53* were conditionally targeted. Gene expression analyses revealed that all tumors expressed a signature strongly associated with the Proneural subgroup which is consistent with *PDGFRA* amplified tumors in adults [29, 31]. The authors also used the mouse HGA expression profiles to identify two groups of patients with Proneural tumors with significantly different survival outcomes [61].

These studies demonstrate that GEMM HGAs can recapitulate not only tumor histology accurately but also their molecular pathology. Depending on their design, the models can replicate a spectrum of human HGA subtypes or phenocopy specific ones [42, 61]. As GEMMs are increasingly incorporated into preclinical therapeutic trials [62], a comprehensive understanding of each one's molecular characteristics will be required to translate the results into effective clinical trial designs.

## USING GEMMS TO TRACE THE CELL-OF-ORIGIN OF HGAS

GEMMs offer a unique opportunity to analyze the early events of gliomagenesis which cannot be accurately determined in studies of human tumors or with xenograft models. Careful analysis of early time points in these mouse models coupled with clever tools to trace transformed cells has resulted in a refined understanding of the cell-of-origin of astrocytomas. By analyzing cells that accumulate mutant p53 soon after Cre-mediated recombination, Wang *et al*. suggest that HGAs in their model originate from the Gfap-positive neural precursor cell (NPC) in the subventricular zone and/or from Olig2-expressing progenitor-like cells most commonly found in the corpus callosum [46].

However, a recent report utilized the same *GFAP-Cre* driver line in conjunction with an elegant genetic tag to label cells that had undergone homozygous deletion of *Trp53* and *Nf1* [63]. These investigators demonstrated that while tumor suppressor deletion occurred in the NPC, these cells remained quiescent until after differentiation into oligodendrocyte precursor cells (OPCs) when they acquired a proliferative phenotype associated with cellular transformation. Similarly, the OPC was also implicated as the transforming cell type in experiments where mutant cells were retrovirally labeled [61]. Interestingly, in the latter study, viral injections were targeted to subcortical white matter and not to the subventricular zone. Clearly, GEMMs using both virally delivered Cre and mouse Cre-expressing lines have demonstrated that HGAs can arise outside of the canonical adult neural progenitor cell niches [42, 47, 48]. The nature of the tumor initiating cell has yet to be rigorously investigated in these models.

These results highlight the differences in experimental approach between the various GEMMs and what we can learn from their use (Table 1). Targeted delivery of viruses to specific brain regions can be used to label relatively few cells and may be an ideal approach to identify and study certain tumor initiating cells. However, tumors initiating from deep seated regions of the brain or from less common sites or cell types may not be efficiently targeted by this approach. The use of Cre mouse lines like *GFAP-Cre* that have activity in the vast pool of embryonic neural stem cells allow for the study of early events of tumorigenesis in these cells and their derivatives. Due to this embryonic activity, there is a strong bias in these models for transformation of the embryonic neural stem cell population over more mature cell types. The *GFAP-CreER* driver line allows for widespread and unbiased genetic manipulation in mature astrocytes and progenitor cells from all regions of the central nervous system and has revealed tumor formation in non-proliferative zones such as the cortex, brain stem, cerebellum and spinal cord. It is important to note that human HGAs arise predominantly in these non-proliferative regions of the brain. However, identifying the cell-of-origin in these models is challenging and will require additional genetic markers. Further study of these different GEMMs will continue to contribute to our understanding of HGA initiation and progression.

## THE PROSPECTS FOR HGA GEMMS TO IMPACT PATIENT OUTCOME

**Figure 2:**
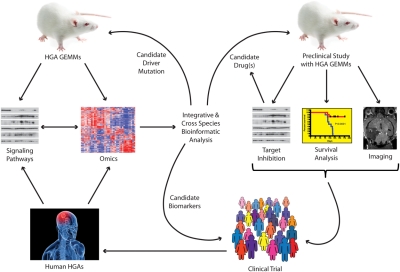
Framework for translational research using genetically engineered mouse models (GEMMs) for high-grade astrocytoma (HGA) Tumors arising in HGA GEMMs are subjected to genome-wide analyses (array comparative genomic hybridization, gene expression, microRNA expression). The status of signaling pathways are determined by western blots or immunohistochemistry. These data are analyzed integratively and can be compared to similar data obtained from human HGAs to identify critical driver mutations, pathways and biomarkers of disease. Candidate genes can be validated in novel GEMMs while biomarkers can be queried either retrospectively or prospectively in clinical trials. Identified pathways with known inhibitors can be tested in the GEMMs using various readouts of efficacy. Promising agents or combinations are carried forward into clinical trials. These in turn will lead to banking and further studies of HGA tumor samples.

HGA continues to be one of the most challenging malignancies that patients and clinicians face. It is clear that substantial improvements in neurosurgical techniques, perioperative care and delivery of radiotherapy have failed to appreciably improve patient outcome [57]. Likewise, conventional chemotherapy has only succeeded in producing a modest clinical impact. As molecularly targeted therapy continues to make inroads in the practice of contemporary clinical oncology, it is anticipated that these approaches will also prove beneficial for HGA. Preclinical investigations will have maximal impact if they employ a series of molecularly characterized animal models that are collectively representative of gene expression and pathway mutation subgroups (Figure 2). Only by systematically testing new molecularly targeted agents against the panel of models will we understand how pharmacologic pathway manipulations differentially impact the biology of HGA subgroups. Such studies could be facilitated by increased cooperation between industry and academia as well as between different investigators working with GEMMs. We anticipate that the knowledge gained from such efforts will help predict which combinations of agents may be beneficial for specific patient subgroups and lead to clinical trials testing these concepts.

Perhaps more importantly, comparative and cross-species analyses of tumors arising from the various models will help elucidate biological details about HGAs that will be informative for patient selection and stratification during these trials (Figure 2). Future investigations may address how mutations at multiple nodes of a pathway cooperate during gliomagenesis, the contribution of frequent HGA-associated mutations, such as IDH1 R132 which has not been modeled to date, and the identification of biomarkers of disease that can be used for predicting response to treatment and to follow tumor burden. GEMMs represent powerful tools with the potential to elucidate HGA biology and to inform the next generation of clinical trials. The judicious use of multiple models will accelerate these discoveries and offer hope to HGA patients who continue their fight for survival against vanishing odds.
